# Co-delivery of endometrial mesenchymal stem cells and macrophages by an electrospun patch promotes angiogenesis during endometrial injury repair via VEGF related signalling

**DOI:** 10.1186/s13287-026-04929-2

**Published:** 2026-02-16

**Authors:** Jiangru An, Shuhong Li, Tianyi Ma, Yonghua Chen, J. Paul Santerre, Wenshuang Wang, Xiaoqing Zhang

**Affiliations:** 1 International Joint Laboratory of Biomaterials and Tissue Regeneration, School of Basic Medicine, Binzhou Medical University, Yantai, 264003 Shandong China; 2 Department of Obstetrics, Yuhuangding Hospital, Yantai, 264000 Shandong China; 3 Faculty of Dentistry and Institute of Biomedical Engineering, University of Toronto, Toronto, ON M5G 1M1 Canada; 4 Li Ka Shing Faculty of Medicine, The University of Hong Kong, Pokfulam, 999077 Hong Kong SAR China; 5 Department of Gynecology, Yuhuangding Hospital, Yantai, 264000 Shandong China

**Keywords:** Co-delivery, Electrospun patch, Intrauterine adhesion, Angiogenesis, Macrophages

## Abstract

**Background:**

Intrauterine adhesion (IUA) is a common gynecological disease that contributes to infertility. Decreased endometrial angiogenesis and uterine ischemia are major therapeutic challenges for IUA and cannot be addressed by current treatment strategies. Human endometrial mesenchymal stem cells (H-EMSCs) and macrophages (mø) are both important cell types that reside within the endometrial tissue and participate in its repair and regeneration. However, how to harness the endometrial tissue repair potential of H-EMSCs and mø simultaneously in a co-delivery system and whether there are significant biochemical cross-talks between the two cell types so that they can regulate each other to specifically boost endometrial tissue angiogenesis remains to be explored.

**Methods:**

This study developed a H-EMSCs-mø co-delivery system using an electrospun polycaprolactone-hyaluronic acid (PCL-HA) membrane and established a rat endometrial damage model. The effects of the co-delivery system on endometrial tissue repair (endometrium thickness, endometrial glands number) and angiogenesis were investigated. The mechanisms underlying the enhanced endometrial tissue angiogenesis of the H-EMSCs-mø co-delivery system were also delineated. All data were analyzed using analysis of variance with Tukey’s test for pair-wise comparisons or an independent samples t-test where appropriate.

**Results:**

In this study, it was found that a H-EMSCs and mø co-delivery system developed with a PCL-HA electrospun membrane carrier (PCL-HA/H-E/mø) significantly increased the endometrium thickness and restored the number of endometrial glands at day 7 and 14 in the endometrial damage model vs. the NR (normal repair) and PCL-HA alone groups. Further, PCL-HA/H-E/mø enhanced more CD31 gene and protein expression, indicating great potential for angiogenesis to occur at day 7 and 14 post-implantation, when compared with PCL-HA/H-E, NR or PCL-HA alone. It was also proved to demonstrated that elevated VEGF production was one of the potential factors that contributed to the enhanced angiogenesis of the co-delivery patch system.

**Conclusions:**

This study provided significant insights into the use of co-delivered H-EMSCs and mø, on a PCL-HA hybrid electrospun membrane, for effectively inducing endometrial angiogenesis and repair to enhance IUA treatment outcomes.

**Supplementary Information:**

The online version contains supplementary material available at 10.1186/s13287-026-04929-2.

## Highlights

This study developed a H-EMSCs and mø co-delivery system (PCL-HA/H-E/mø)

PCL-HA/H-E/mø increased endometrium thickness and restored glands number

PCL-HA/H-E/mø enhanced CD31 gene and protein expression showing more angiogenesis

Elevated VEGF production could have caused angiogenesis in PCL-HA/H-E/mø system

PCL-HA/H-E/mø hybrid system induced endometrial angiogenesis and could treat IUAs

**Supplementary Information:**

The online version contains supplementary material available at 10.1186/s13287-026-04929-2.

## Background

Intrauterine adhesion (IUA), which is also known as the Asherman’s syndrome, is a common gynecological disease condition characterized by aberrant adhesions within the uterus that cause partial or complete obliteration of the uterine cavity [1]. Approximately 90% of IUAs were caused by intrauterine surgery or infection, with extensive damage to the regenerative basal layer of the endometrium [2]. Such endometrial basal layer damage can further lead to inflammation, impaired epithelial cell regeneration and decreased angiogenesis, causing uterine ischemia that represents a major therapeutic challenge [3, 4]. Currently, the treatment strategies for IUAs primarily include hysteroscopic lysis of adhesions, hormonal therapy, or physical barriers (e.g., amniotic membranes, balloons and intrauterine devices) [1, 3]. However, the effectiveness of those therapeutic strategies is low, as they rely on the endometrium’s self-repair capacity, rather than fundamentally improving the pathological micro-environment of the injured endometrium. Recent studies elucidating the pathogenesis of IUAs have suggested that intervening in the inflammatory pathways of the endometrial micro-environment and simultaneously enhancing endometrial angiogenesis, would be crucially important towards effectively treating IUAs [5, 6]. However, methods to improve the inflammatory endometrial micro-environment and enhance endometrial angiogenesis require further exploration.

Angiogenesis, which refers to the development of new capillaries from pre-existing micro-vessels, is a critical step in menstrual wound healing and contributes to a series of ovulatory-related and non-ovulatory-related reproduction processes [5, 7]. Previous studies showed that abnormal angiogenesis was strongly associated with reduced decidualization, and it could be one of the significant causes of recurrent pregnancy loss [5]. Additionally, disruptions of angiogenesis can negatively affect the normal healing of uterine scars [8]. As a result, therapeutic strategies that can promote endometrial tissue angiogenesis are desired to effectively treat IUAs.

Studies have shown that human endometrial mesenchymal stem cells (H-EMSCs) have self-renewal, immunomodulation capabilities and can directly repair the damaged endometrium by promoting new endometrial tissue formation and increasing endometrial thickness [9, 10]. During the menstrual cycle, it was also found that H-EMSCs can induce endometrial tissue angiogenesis via the activation of the serine–threonine kinase AKT and extracellular signal-regulated kinase (ERK) signalling pathways [11]. Moreover, other local cells associated with injury, specifically monocyte-derived macrophages (mø), were demonstrated to play a fundamental role in endometrial tissue repair and regeneration after injury [12]. Mø are well-acknowledged to be key regulators of tissue inflammation, fibrosis, remodelling and regeneration [13]. In the process of endometrial tissue repair, mø can remove endometrial tissue debris through phagocytosis and secrete a series of pro-inflammatory and anti-inflammatory cytokines and growth factors [12]. Furthermore, in vivo studies showed that mø could potentially enhance angiogenesis in endometrial tissue repair and regeneration [14–16], and the deletion of mø suppressed endometrial tissue angiogenesis and caused defective development of very thin endometrium [14]. Although H-EMSCs and mø could both participate in endometrial repair and regeneration, and there could be significant cross-talk between the two [17], there are currently no studies that have investigated strategies to co-deliver H-EMSCs and mø simultaneously, in a manner that the cells were provided an opportunity to establish (prior to implantation) vital biomolecular cross-talk in a co-culture system, with the end-goal to enhance endometrial injury repair and specifically promote endometrial angiogenesis.

The direct injection of mesenchymal stem cells (MSCs) has been reported to have some effects for endometrial tissue repair [18]. However, such intervention could not be sustained for a prolonged period of time because of the low survival rate and short retention time of the transplanted MSCs at the implant site, due to hypoxia and nutrient deficiency in the damaged endometrial tissue micro-environment [19]. Biocompatible biomaterials, which can mimic the structure of the natural extracellular matrix (ECM), could provide an appropriate micro-environment for cell attachment, proliferation, migration [20]. Therefore, biomaterials can serve as not only a scaffold but also a delivery vehicle for the MSCs, to potentially promote endometrial tissue repair and regeneration [20]. However, the caveat is that the biomaterial itself must be formulated and processed in a manner that it does not itself, elicit an overwhelming foreign body response [21]. Polycaprolactone **(**PCL) is a well-known polymer approved by the U.S. Food and Drug Administration (FDA) and has been shown to have good biocompatibility, controlled biodegradability, as well as strong mechanical properties, qualifying it as a potential cell carrier for soft tissue engineering applications [22]. However, if presented to the wound site in an inappropriate processed form, PCL can induce a pronounced foreign body response [23]. Hence, other co-processed biomaterials should be considered for use with PCL. For example, hyaluronic acid (HA), which is an anionic linear polysaccharide and a critical component of the natural ECM, has been demonstrated to have excellent hydrophilicity, biodegradability, biocompatibility and non-immunoreactivity [24]. Recently, our research group has developed a PCL-HA patch (PCL: HA = 80:20) via electrospinning technique and it demonstrated ultrafine continuous fibers, high porosity, high surface-to-volume ratios, in a manner that structurally mimicked the native ECM [25]. This PCL-HA electrospun membrane supported H-EMSCs’ attachment, proliferation, enhanced the H-EMSCs**’** expression of wound-healing genes IL-10, VEGFA, TGF-β but suppressed their expression of the tissue inflammation gene IL-6 [25]. In addition, PCL-HA also supported MSC markers CD90 and Meflin expression of the seeded H-EMSCs [25]. All of our previous findings have established PCL-HA as a model biomaterial platform for endometrial tissue repair and IUA treatment.

Consequently, this study aimed to develop an endometrial tissue injury model, and determine if PCL-HA electrospun membranes, co-delivered with H-EMSCs and mø could establish a vital pre-formed ECM and biomolecular cross-talk state which would improve endometrial thickness, increase the number of endometrial glands and promote endometrial angiogenesis. The work also delineated some of the angiogenic mechanisms involved in the interactions between H-EMSCs and mø when co-delivered with PCL-HA. This study would provide significant insights into the feasibility of using a co-delivery system, such as PCL-HA/H-EMSCs/mø, to promote endometrial angiogenesis, effectively repair endometrial injury and potentially treat IUAs.

## Methods

All chemicals were purchased from Solarbio Life Sciences and used as is unless stated otherwise. The work has been reported in line with the ARRIVE guidelines 2.0.

### Isolation and culture of H-EMSCs

Human endometrial tissue pieces (released into menstrual blood) were obtained from the Yuhuangding Hospital of Yantai (ethics approval number: 2023-043). 15 ml PBS (P1020, Solarbio) containing 1% penicillin/streptomycin (PB180120, Procell) was added to the 50 ml centrifuge tube and the human endometrial tissue samples were collected in the tube and transported on ice. The endometrial tissue was rinsed with PBS, cut into 1 mm³ pieces and digested with collagenase type I (1 mg/ml, BS163, Biosharp) for 60 min at 37℃ within a constant temperature oscillator (80 rpm). DMEM/F12 complete medium (PM150312, Procell, containing 10% FBS (164210, Procell) and 1% penicillin/streptomycin) was used to terminate the digestion. The cells were centrifuged at 1000 rpm for 5 min and the cell pellet was re-suspended with DMEM/F12 medium and seeded into T75 TCPS culture flasks to obtain passage 1 H-EMSCs. Culture medium was changed 24 h immediately after seeding and then changed every 2–3 days in culture. When H-EMSCs reached 80%-90% confluency, they were passaged using a ratio of 1:2. P4 H-EMSCs were used for all the experiments in this study, according to previous studies [26–28].

The clinical information of patients who donated endometrial tissue samples of the study can be found in the Supplemental Table 1 below. Characterization of the isolated H-EMSCs including (morphology, cell doubling time, flow cytometry for CD90, CD73 and CD45, colony forming capability, adipogenic, osteogenic and chondrogenic differentiation) was already performed in a previous study carried by our group [25], so it was not repeated in the current study. The cells extracted from separate donors after separating the mesenchymal cells were pooled and used for the experiments.

### Fabrication of PCL-HA electrospun carriers

PCL-HA electrospun carriers were fabricated according to our previously established protocol [25]. Specifically, PCL: HA (80:20) were added to 10 mL hexafluoro-isopropanol and the solution was magnetically stirred for 12 h to obtain a transparent spinning solution. The solution was poured into a syringe and the electrospinner (Ne300, Inovenso Inc.) was set to have a flow rate of 1.5 mL/h, a spinning voltage of 12.6 kV and a spinning distance of 20 cm. After electrospinning, the 2.5 cm×0.5 cm PCL-HA electrospun carriers were dried in a vacuum oven for 48 h to remove residual solvent and then stored in a desiccator in the dark until use. Characterization studies of PCL-HA (i.e., scanning electron microscopy, fiber diameter, material porosity % and degradation rate, as well as the material biocompatibility (the viability, proliferation and metabolic activities of the H-EMSCs)) were already carried out in our previous research [25], so those were not repeated in the current study.

### H-EMSCs mono-delivery and H-EMSCs/mø co-delivery with PCL-HA carriers

PCL-HA electrospun carriers were placed into tissue culture discs and sterilized with 70% ethanol (overnight) and then PBS was added to remove all residual ethanol. 1 × 10^6^ H-EMSCs suspended in 50 µl medium or 1 × 10^6^ H-EMSCs + 0.25 × 10^6^ mø (isolated from human peripheral blood according to our lab’s previous protocol [29, 30]) suspended in 50 µl medium were seeded onto the PCL-HA electrospun carriers for H-EMSCs mono-delivery and H-EMSCs/mø co-delivery conditions, respectively. The H-EMSCs: Mø ratio in the co-delivery condition was determined based on our group’s previously published studies [31, 32]. The cell-loaded PCL-HA electrospun carriers were implanted into the rat endometrial damage model 24 h post-cell loading.

### Establishment of a rat endometrial damage model and implantation of PCL-HA carriers

Female Sprague-Dawley (SD) rats (220–250 g, 8–10 weeks old) were purchased from Jinan Pengyue Biotechnology Co., Ltd. The rats were maintained at 22 °C with 12 h/12 h light and dark cycle and served with adequate food and water before the experiments. Ninety estrus female SD rats were divided into five groups: Sham operation group, natural repair group (NR) (i.e., normal repair, no intervention), PCL-HA group, PCL-HA/H-E group and PCL-HA/H-E/mø group (H-E and mø stands for H-EMSCs and macrophages, respectively), with three time points (day 3, day 7 and day 14, 6 rats/time point) for each group. All rats were anesthetized by intraperitoneal injection of pentobarbital sodium (30 mg/kg), shaved in the middle and lower abdominal wall, and sprayed with 75% ethanol to disinfect the operation area. The rats’ abdominal wall was cut layer by layer to fully expose the bilateral uterine horn and a small incision was made near the fallopian tube of the uterus. A 16G syringe needle was inserted to scratch the uterine wall 8–10 times until the uterine surface became rough and bleeding, to establish the physical endometrial damage model. After flushing with PBS, the PCL-HA, PCL-HA/H-E and PCL-HA/H-E/mø carriers were implanted to the surface of the damaged endometrium and the uterine wall incision was closed with 6 − 0 absorbable sutures.

For the VEGF inhibition experiments, 18 female SD rats in estrus were divided into the following three groups, with 6 rats in each group: PCL-HA/H-E group: PCL-HA electrospun carrier (1 × 10^6^ H-EMSCs) was transplanted; PCL-HA/H-E/mø group: PCL-HA electrospun carrier (1 × 10^6^ H-EMSCs + 0.25 × 10^6^ mø) was transplanted; PCL-HA/H-E/mø/VEGF inhibitor group: PCL-HA electrospun carrier (1 × 10^6^ H-EMSCs and 0.25 × 10^6^ mø) was implanted, VEGF inhibitor (Axitinib (HY-10065, MCE), 100 mg/kg) was injected into the rat uterine cavity.

Intraperitoneal injection of 200 mg/kg pentobarbital sodium was used for euthanasia of the rats in this study.

### Collection of endometrial tissue samples and preparation of paraffin and frozen sections

At days 3, 7 and 14, the rats in each group were euthanized and endometrial tissue samples were collected. Specifically, a longitudinal incision was made along the uterine segment and then it was spread out flat. After that, the endometrial layer and the underlying muscularis were separated gently according to the junctions between the two layers. The tissue samples were washed with PBS and fixed with 4% paraformaldehyde (BL539A, Biosharp) at 4 °C for 48 h. For the VEGF inhibition experiment, the samples were studied at day 7. Subsequently, paraffin and frozen sections were prepared for the histology (H&E staining) and immunofluorescence staining experiments of the study, respectively. For qRT-PCR experiments, the endometrial tissues were collected and stored in liquid nitrogen until needed.

For the paraffin section preparation, the fixed endometrial tissues were cut into 3 mm-long segments, dehydrated and embedded with the following process: 75% ethanol (2 h) →85% ethanol (2 h) →95% ethanol (1 h x2) →100% ethanol (1 h x2) →xylene (30 min x2) →wax (1 h) →embedding. The endometrial tissue samples were cut to a thickness of 5 μm using a microtome (Leica Biosystems Inc). Deparaffinization was achieved using the following procedure: xylene (15 min x2) →100% ethanol (10 min x2) →95% ethanol (5 min x2) →85% ethanol (5 min) →75% ethanol (5 min) →distilled water (1 min).

For the frozen section preparation, the fixed tissue samples were dehydrated in 30% sucrose for 48 h. Then, tissue-Tek O.C.T. frozen section embedding medium (4583, Sakura Finetek Japan Co., Ltd.) was used to embed the endometrial tissue samples. A Thermo Scientific™ CryoStar™ NX50 Cryostat was used to obtain frozen tissue sections with a 10 μm thickness.

### H&E staining

The endometrial tissue sections were firstly placed in hematoxylin (IH-017B, Novland) and stained for 10 min, then they were washed with distilled water for 1 min, soaked in 1% hydrochloric acid alcohol for 3s, and subsequently flushed with tap water for 10 min. Afterwards, the tissue sections were immersed in eosin staining solution (IH-018B, Novland) for 5 min and treated with ethanol gradients (75%, 85%, 95% (5 min each), 100% ethanol (5 min x2)). Then, the tissue sections were treated with xylene (5 min x2) and sealed with neutral balsam. Endometrial tissue sections were observed under an inverted histology microscope (Zeiss). The endometrial layer thickness and number of endometrial glands were analyzed using the ruler tool and counting tool in Image J (Version 1.54).

### Immunofluorescence

The frozen sections were soaked in PBS for 10 min to remove the tissue-Tek O.C.T. frozen section embedding medium. 10% goat serum (SL038, Solarbio) was used for blocking (room temperature, 1 h) and the sections were treated with primary antibody solution at 4 °C for overnight. The next day, the tissue sections were incubated at room temperature for 1 h and washed with PBS (5 min x3). The sections were then treated with secondary antibody solutions for 1 h in the dark at room temperature. Cell nuclei were counter-stained with DAPI (10 µg/ml, C0065, Solarbio) (room temperature, 10 min in the dark), and anti-fade mountant (S2100, Solarbio) was added to all tissue sections before they were imaged with a Nikon upright fluorescence microscope. The following lists the primary and secondary antibodies used: rabbit polyclonal to CD31 (1:200, 28083-1-AP, Proteintech); rabbit polyclonal to VEGF (1:200, PB9071, Boster); AlexaFluor^®^ 488 goat anti-mouse IgG (1:200, A28175, Thermo scientific) and AlexaFluor^®^ 568 goat anti-rabbit IgG (1:200, A-11011, Thermo scientific). Immunofluorescence images were analyzed using Image J (Version 1.54) according to our previously established procedures [31, 32].

### Tube formation assay

Cell culture supernatant was collected from H-EMSCs mono-delivered (5 × 10^4^ cells) or H-EMSCs/mø co-delivered (5 × 10^4^ H-EMSCs + 1.25 × 10^4^ mø) on PCL-HA electrospun carriers after culturing for 24 h. Matrigel capillary formation assay (356234, BD Biosciences) was used to evaluate the effects of H-EMSCs mono-delivery or H-EMSCs/mø co-delivery (on PCL-HA) on angiogenesis in vitro. Human umbilical vein endothelial cells (HUVECs) were seeded on Matrigel coated 96-well plates with a density of 30,000 cells/well, and the cells were treated with supernatants collected from H-EMSCs mono-delivered or H-EMSCs/mø co-delivered on PCL-HA. For the PCL-HA/H-E/mø/VEGF inhibitor group, HUVECs were treated with supernatant collected from H-EMSCs/mø co-delivered on PCL-HA and VEGF inhibitor Aflibercept (IA8041, Solarbio, 500 µg/mL, according to the manufacturer’s instructions). The formation of the tubes was examined by microscopy (Zeiss) after 6 h of culture. The total lengths of the tubes formed, the number of meshes, as well as the nodes formation were analyzed using ImageJ’s ruler and counting tools.

### qRT-PCR

mRNA was extracted from the cells or endometrial tissues using the Trizol (R401-01, Vazyme) method. mRNA quality and quantity were checked with NanoDrop™ 1000 Spectrophotometer (Thermo Scientific), and the samples were stored in a -80 °C freezer. cDNA was synthesized using the reverse transcription kit (R233-01, Vazyme), according to the manufacturer’s protocol. The obtained cDNA samples were diluted 10 times with nuclease free water and used for qRT-PCR. Reaction mixtures containing 10 µl 2 x ChamQ SYBR qPCR Master Mix (Q311-02, Vazyme), 0.4 µl forward primer (10 µM), 0.4 µl reverse primer (10 µM), 0.7 µl cDNA sample, 8.5 µl ddH_2_O were prepared. The qPCR reaction was performed with Roche LightCycler™ Real-Time PCR Detection System, using the following protocol: Pre-incubation: 95 °C for 10 min, Amplification (40 cycles): 95 °C for 10 s, 60 °C for 30 s, Melting curve: 95 °C for 15 s, 60 °C for 1 min, 95 °C for 15 s. The data was analyzed using the comparative 2^−ΔΔCt^ method. The forward and reverse primer sequences of the genes can be found in the following Table 1.

For the VEGF inhibition experiment, the cells included the following groups: Control group: 5 × 10^4^ HUVECs treated with DMEM high-glucose medium; PCL-HA/H-E group: 5 × 10^4^ HUVECs treated with cell culture supernatant of H-EMSCs seeded on PCL-HA; PCL-HA/H-E/mø group: 5 × 10^4^ HUVECs treated with cell culture supernatant of H-EMSCs/mø co-seeded on PCL-HA; PCL-HA/H-E/mø/VEGF inhibitor group: 5 × 10^4^ HUVECs treated with cell culture supernatant of H-EMSCs/mø co-seeded on PCL-HA and VEGF inhibitor Aflibercept (500 µg/mL). The cells were treated for 24 h before mRNA extraction.

**Table 1:** The forward and reverse primer sequences of the genes (homo-VEGF, homo-CD31, rat-VEGF, rat-CD31)

homo-VEGF	Forward (5′–3′): GGGCAGAATCATCACGAAGTGG Reverse (5′–3′): AAGATGTCCACCAGGGTCTCG
homo-CD31	Forward (5′–3′): ACGTGCAGTACACGGAAGTTCA Reverse (5′–3′): AGGGAGCCTTCCGTTCTAGAGT
rat-VEGF	Forward (5′–3′): CGTCCAACTTCTGGGCTCTTC Reverse (5′–3′): AGCACTTCTCCCAGCTCCGAT
rat-CD31	Forward (5′–3′): ACGTGCAGTACACGGAAGTTCA Reverse (5′–3′): AGGGAGCCTTCCGTTCTAGAGT

### ELISA

A longitudinal incision was made along the uterine segment and then it was spread out flat. Afterwards, the endometrial layer and the underlying muscularis were separated gently according to the junctions between the two layers. Approximately 0.5 g of endometrial tissue samples were collected and washed with pre-cooled PBS to remove blood, then the tissues were minced with ophthalmic scissors, homogenized using a tissue homogenizer, and finally the tissue samples were transferred to a 1.5 ml centrifuge tube containing 200 µl PBS and centrifuged at 11,200 rpm for 15 min at 4℃ and stored on ice. The protein concentrations of the samples were measured using a BCA kit (PC0020, Solarbio) according to the manufacturer’s instructions. The absorbance was measured at 562 nm wavelength using a spectrometer (SpectraMax M2, Molecular Devices, LLC) and the protein concentrations of the samples were calculated and recorded.

For the VEGF quantification of the endometrial tissue homogenates, EliKine™ Rat VEGF ELISA Kit (KTE9008, Abbkine) was used according to the manufacturer’s instructions. Basically, 100 µl diluted standard or sample solution was added to each test well and 100 µl sample diluents were added to the blank wells. Then, 50 µl of the detection antibody solution was added to each well and the plate was sealed and incubated at room temperature for 2 h. After incubation, the plate was washed with 300 µl wash buffer/well for 6 times and 100 µl of streptavidin-HRP was added to each well and incubated for 45 min. Finally, the plate was washed with 300 µl wash buffer/well for 6 times and 100 µl of HRP substrate (TMB) was added to each well and incubated at room temperature in the dark for 15 min. 100 µl of stop solution was added to each well to stop the reaction and the absorbance was taken at 450 nm wavelength using SpectraMax M2 immediately.

### Data analysis

All data were analyzed using SPSS 22.0 software (SPSS Inc., Chicago, IL) by analysis of variance (ANOVA) using Tukey’s test for pair-wise comparisons or an independent samples t-test where appropriate. All experiments were repeated at least three times with at least three samples each time (*N* = 3, *n* = 3) unless stated otherwise. Data was represented as mean ± S.E.M, *p* < 0.05 indicates statistical significance.

## Results

This study established a rat endometrial damage model and probed the effects of a PCL-HA electrospun membrane-based co-delivery system containing H-EMSCs and mø, on enhancing endometrial tissue angiogenesis and promoting endometrial tissue repair (i.e., endometrium thickness, number of endometrial glands). In addition, the regulation of the co-delivery system (PCL-HA/H-E/mø) on angiogenic marker expression (i.e., CD31) and tube formation capability within HUVECs in vitro was investigated. Finally, a prominent signalling pathway underlying the significant promotion of endometrial angiogenesis by the PCL-HA/H-E/mø co-delivery system was discovered.

### PCL-HA/H-E and PCL-HA/H-E/mø systems enhanced endometrial tissue injury repair

**Fig. 1:**
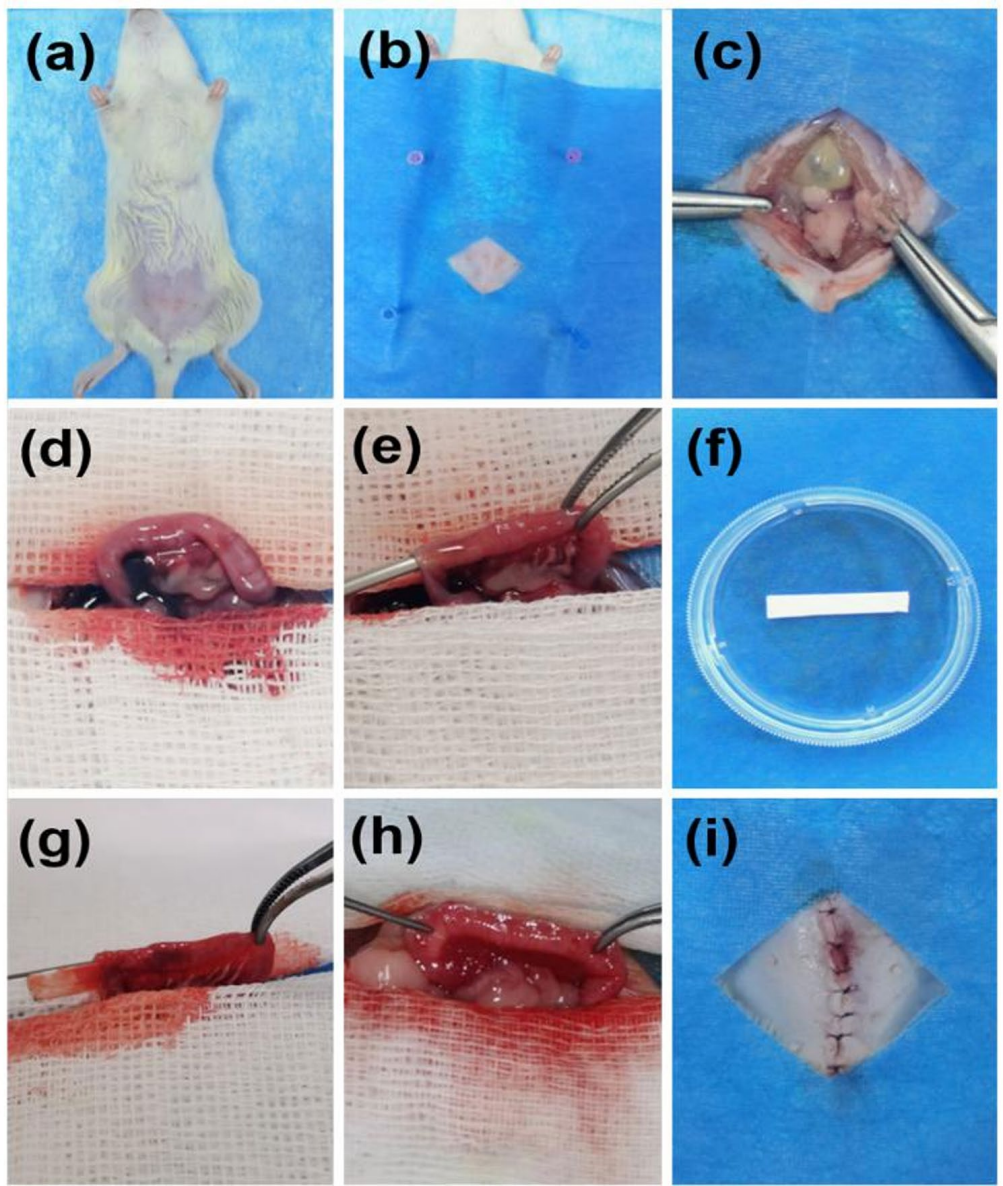
Rat endometrial injury model and implantation of PCL-HA based mono-delivery or co-delivery cell carriers. **a**–**c** After anesthesia, the rat abdomen was dissected to expose the uterine horn. **d**,** e** Creating the endometrial lesion in the rat uterus, with a sterile needle. **f–h** Implanting the PCL-HA based endometrial carriers onto the endometrial lesion area. **i** Closing the wound with absorbable sutures––

**Fig. 2:**
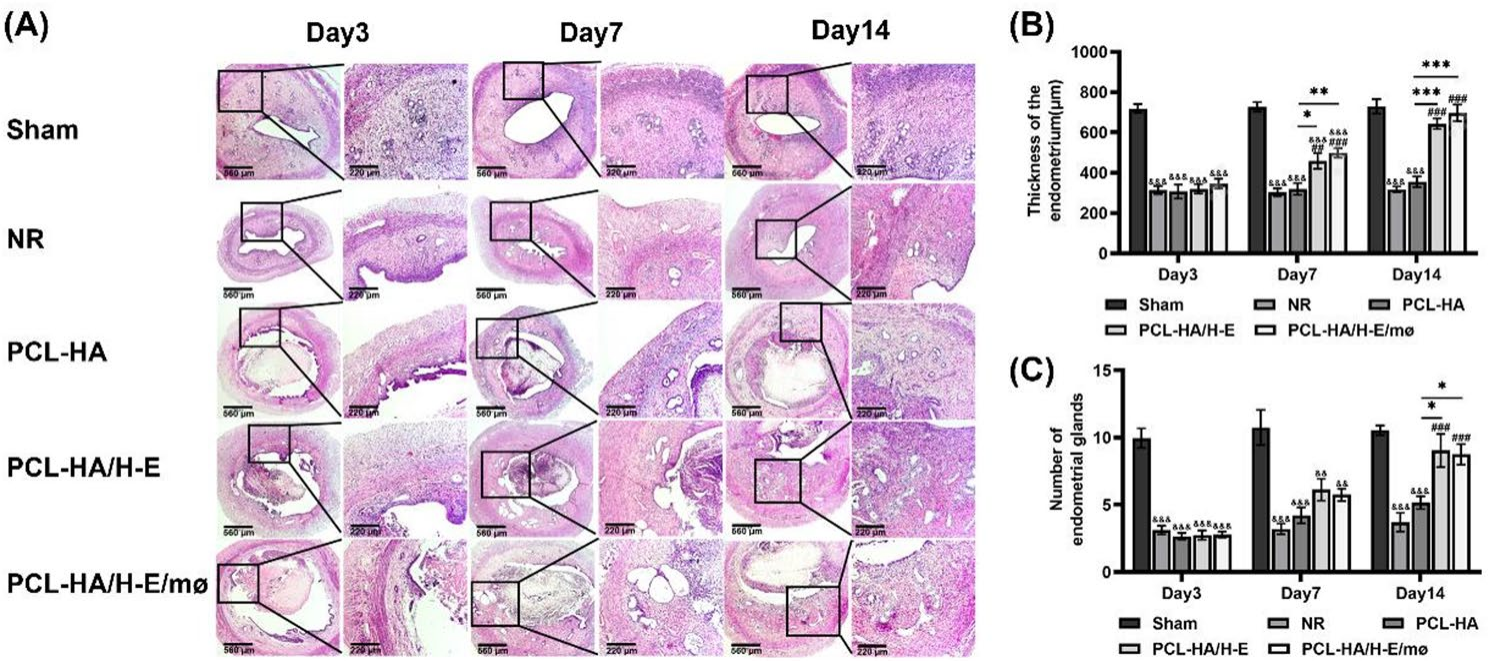
H&E staining of Sham, normal repair (NR), PCL-HA carrier alone, PCL-HA with H-EMSCs mono-delivery (PCL-HA/H-E), PCL-HA with H-EMSCs and mø co-delivery (PCL-HA/H-E/mø) groups, at days 3, 7 and 14. **A** Representative H&E images of the rat uterine tissues of the different treatment groups at days 3, 7 and 14. Scale bar = 560 μm. Enlarged images are shown on the right and scale bar = 220 μm. **B** Quantification of the endometrium thickness at days 3, 7, and 14. **C** Quantification of the number of endometrial glands at days 3, 7 and 14. ^*^p < 0.05, ^**^p < 0.01, ^***^p < 0.001. ^##^*p* < 0.01, ^###^*p* < 0.001 vs. NR. ^&&^*p* < 0.01, ^&&&^*p* < 0.001 vs. Sham

As can be seen in Fig. 1, a rat endometrial injury model was developed and PCL-HA based carriers (PCL-HA carrier alone, PCL-HA carrier mono-delivered with H-EMSCs, PCL-HA carrier co-delivered with both H-EMSCs and mø) were implanted. As can be seen in Fig. 2, comparing the NR (damaged group without any treatment, stands for normal repair) condition with Sham, NR showed significantly lower endometrial thickness and reduced number of endometrial glands. Therefore, the endometrial damage animal model was validated. It was found that the implantations of PCL-HA carrier mono-delivered with H-EMSCs (PCL-HA/H-E) or co-delivered with H-EMSCs and mø (PCL-HA/H-E/mø) both contributed to a significant increase in endometrium thickness at days 7 and 14 in the endometrial damage model vs. the NR and PCL-HA carrier alone (Fig. 2A and B). In addition, implantations of PCL-HA/H-E or PCL-HA/H-E/mø also restored the number of endometrial glands vs. NR or PCL-HA carrier alone (Fig. 2C).

### PCL-HA/H-E/mø enhanced angiogenic marker expression of damaged endometrial tissues

**Fig. 3:**
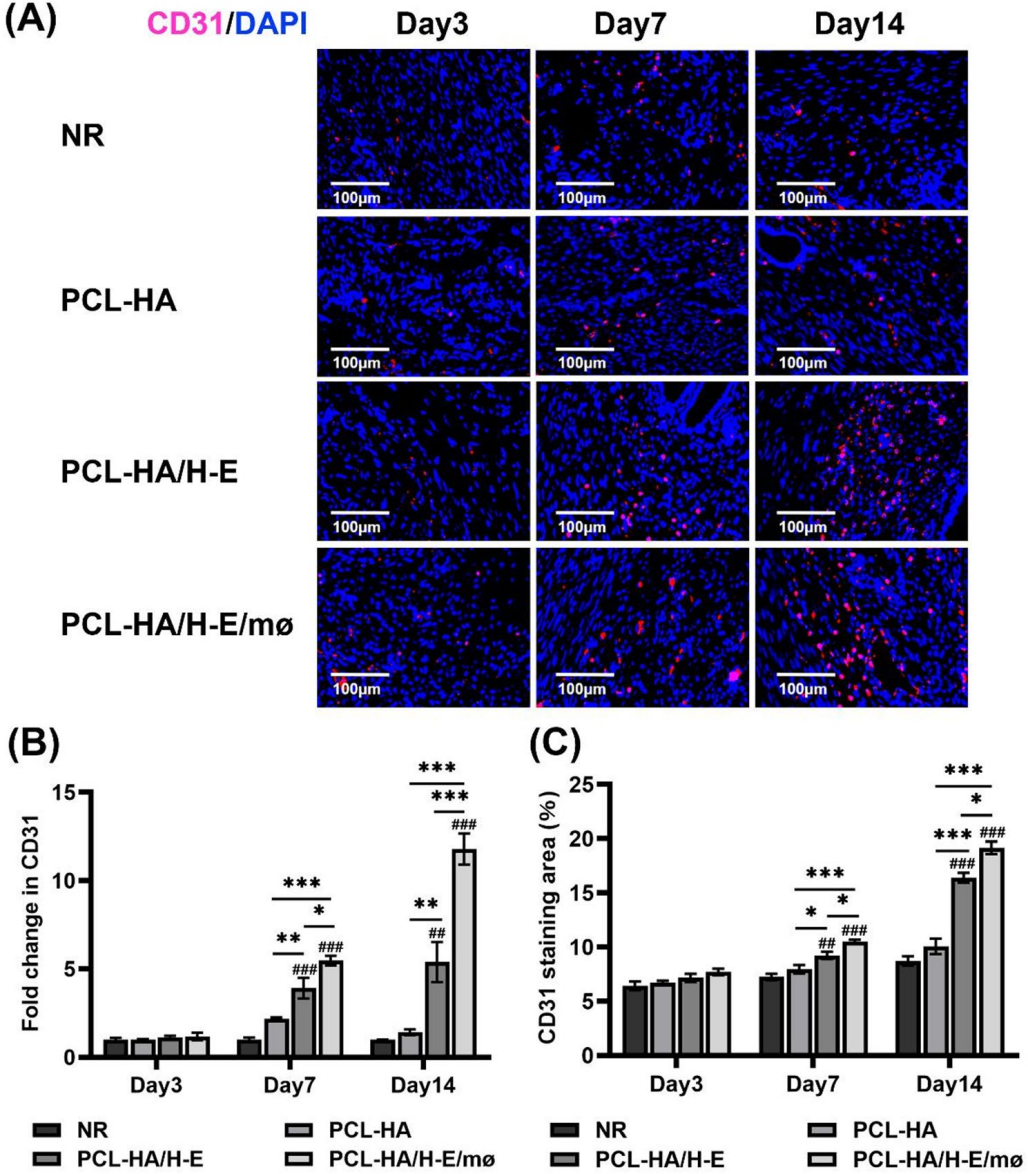
CD31 gene and protein expression in the rat endometrial tissues of the NR, PCL-HA, PCL-HA/H-E, PCL-HA/H-E/mø groups at days 3, 7 and 14. **A** Representative images of CD31 immunostaining of the uterine tissues. The nuclei were stained in blue while CD31 was stained in red. Scale bar = 100 μm. **B** CD31 gene expression in the rat endometrial tissues. ^*^p < 0.05, ^**^p < 0.01, ^***^p < 0.001. ^##^*p* < 0.01, ^###^*p* < 0.001 vs. NR. **C** Quantification of the CD31 immunostaining area (%). ^*^p < 0.05, ^***^p < 0.001. ^##^*p* < 0.01, ^###^*p* < 0.001 vs. NR

Implantations of PCL-HA/H-E and PCL-HA/H-E/mø systems increased CD31 gene and protein expression in the damaged endometrial tissues vs. the NR and PCL-HA groups at days 7 and 14 time points (Fig. 3). Importantly, it was also found that PCL-HA/H-E/mø were more effective in terms of promoting CD31 gene and protein expression vs. PCL-HA/H-E at days 7 and 14 post-implantation (Fig. 3).

**Fig. 4:**
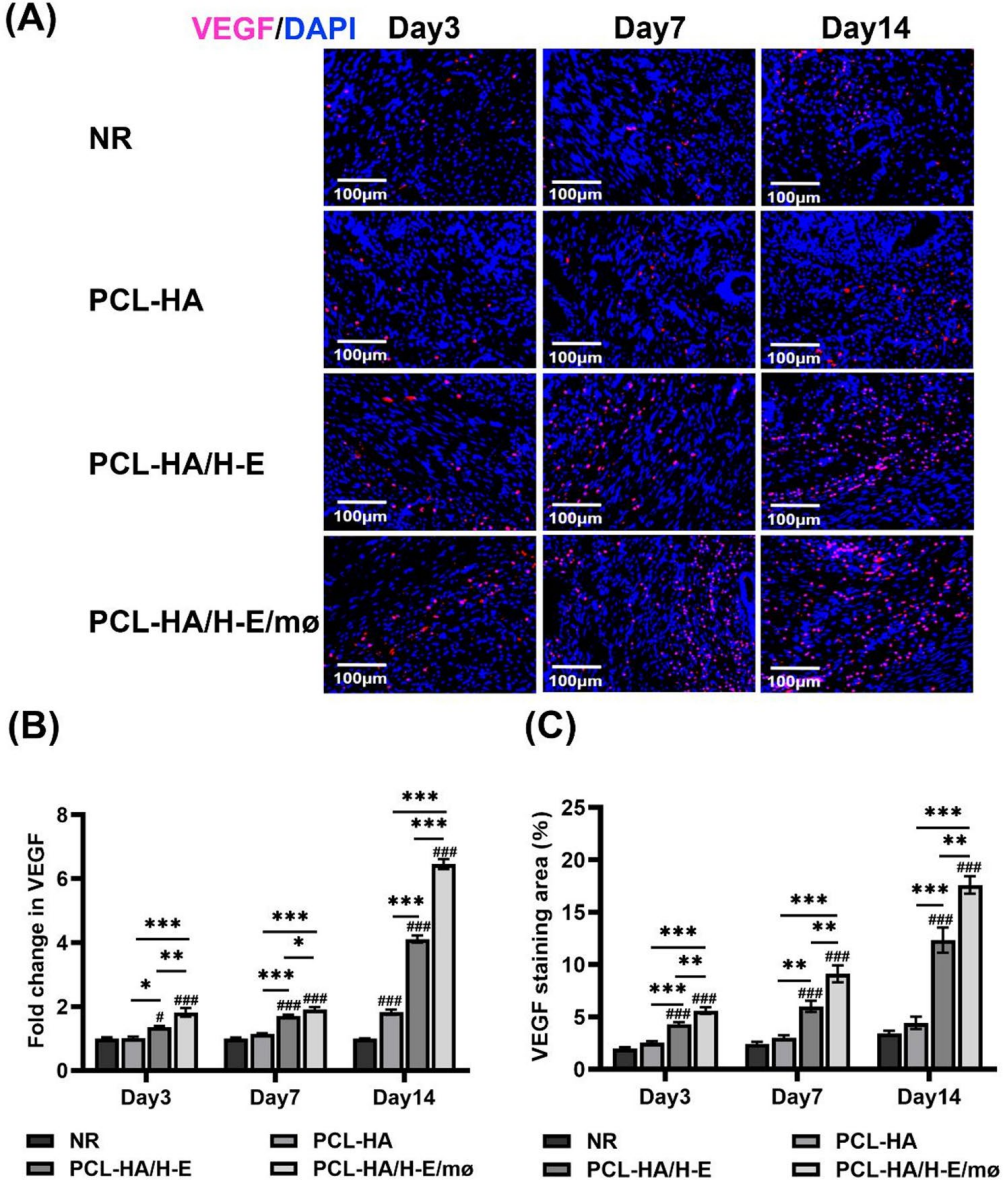
VEGF gene and protein expression in rat endometrial tissues of the NR, PCL-HA, PCL-HA/H-E, PCL-HA/H-E/mø groups at days 3, 7 and 14. **A** Representative images of VEGF immunostaining of the rat uterine tissues. The nuclei were stained in blue while VEGF was stained in red. Scale bar = 100 μm. **B** VEGF gene expression in rat endometrial tissues. ^*^p < 0.05, ^**^p < 0.01, ^***^p < 0.001. ^#^*p* < 0.05, ^###^*p* < 0.001 vs. NR. **C** Quantification of the VEGF immunostaining area (%). ^**^p < 0.01, ^***^p < 0.001. ^###^*p* < 0.001 vs. NR

Both PCL-HA/H-E and PCL-HA/H-E/mø augmented VEGF gene and protein expression of the damaged endometrial tissues vs. the NR and PCL-HA groups, starting from day 3 after implantation (Fig. 4). PCL-HA/H-E/mø also caused higher expression of VEGF at the gene and protein levels in the damaged endometrial tissues, when compared with PCL-HA/H-E at days 3, 7 and 14 time points (Fig. 4).

### In vitro experiments assessing the angiogenic potential of PCL-HA/H-E/mø

According to the results obtained in Sect. 3.1 and 3.2, PCL-HA/H-E/mø system significantly improved the endometrium thickness, enhanced the number of endometrial glands compared to PCL-HA/H-E, and showed higher angiogenic potential in the endometrial damage model (enhanced CD31 gene and protein expression), which also corresponded well with increased VEGF gene and protein expression.

**Fig. 5:**
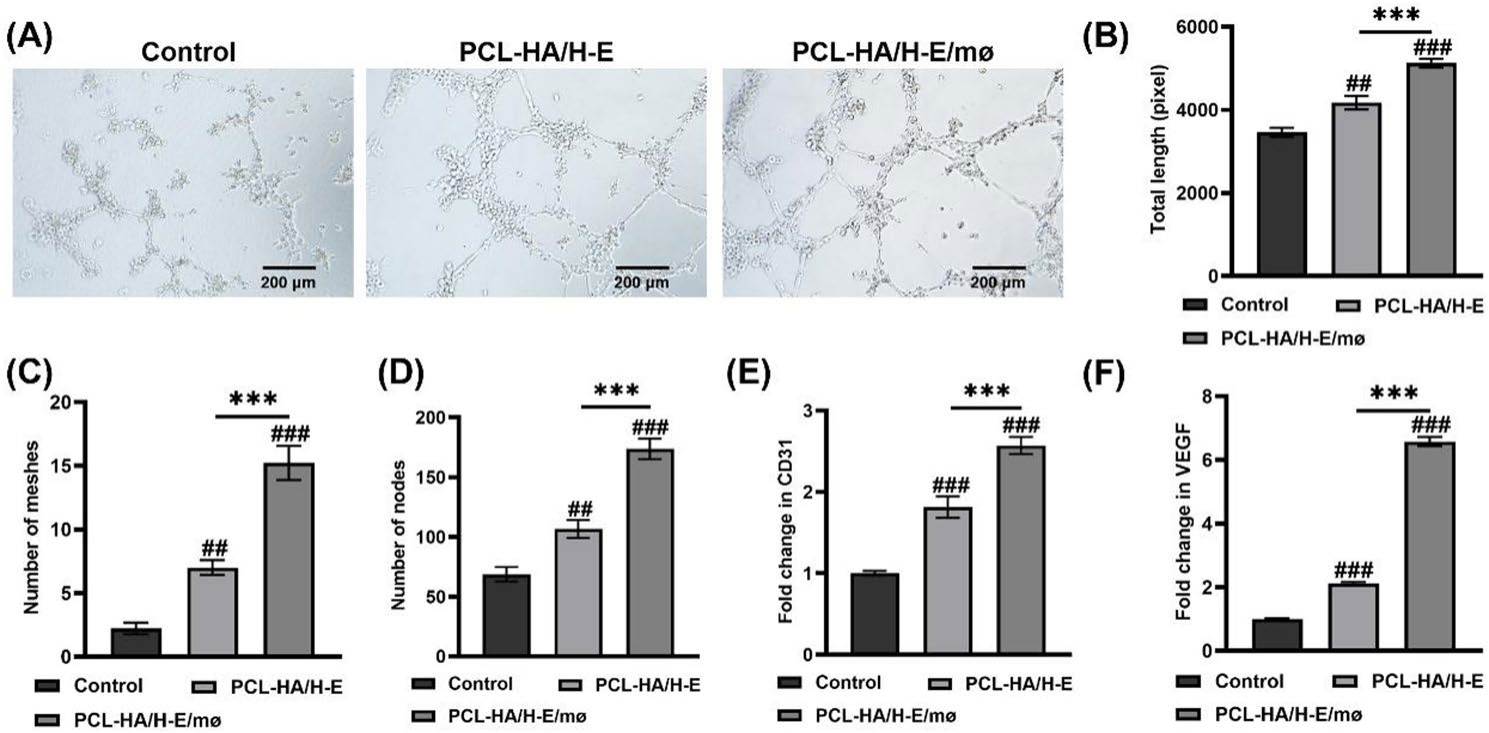
Assessment of the tube formation capability, CD31 and VEGF gene expressions for HUVECs treated without (control) and with cell culture supernatant collected from H-EMSCs seeded on PCL-HA (PCL-HA/H-E) or H-EMSCs and mø co-seeded on PCL-HA (PCL-HA/H-E/mø). **A** Tube formation assay of HUVECs in the control, PCL-HA/H-E and PCL-HA/H-E/mø groups. Scale bar = 200 μm. **B** Quantification of the total length of the tubes formed. **C** Quantification of the total number of meshes formed. **D** Quantification of the number of nodes formed. **E** CD31 gene expression of the HUVECs. **F** VEGF gene expression of the HUVECs. ^***^p < 0.001. ^##^*p* < 0.01, ^###^*p* < 0.001 vs. Control

Previous studies have demonstrated that VEGF can induce the differentiation of functional endothelium from human embryonic stem cells and increase the micro-vessel density (increased CD31 expression) in skin lesions [33, 34]. As a result, it was expected that the promotion of CD31 expression in PCL-HA/H-E/mø condition was associated with the increased VEGF, and hence a tube formation assay was performed to assess the angiogenic potential of PCL-HA/H-E/mø in vitro. As can be seen in Fig. 5A and D, the HUVECs treated with cell culture supernatant collected from H-EMSCs seeded on PCL-HA (PCL-HA/H-E) or H-EMSCs and mø co-seeded on PCL-HA (PCL-HA/H-E/mø) for 24 h, showed greater tube formation capability (increased total tube length, number of meshes and nodes) vs. the control condition. In addition, PCL-HA/H-E/mø condition showed higher angiogenic potential (increased total tube length, number of meshes and nodes) vs. the PCL-HA/H-E condition, which corresponded with higher CD31 and VEGF gene expression in the PCL-HA/H-E/mø condition (Fig. 5E and F).

### VEGF inhibition experiments

**Fig. 6:**
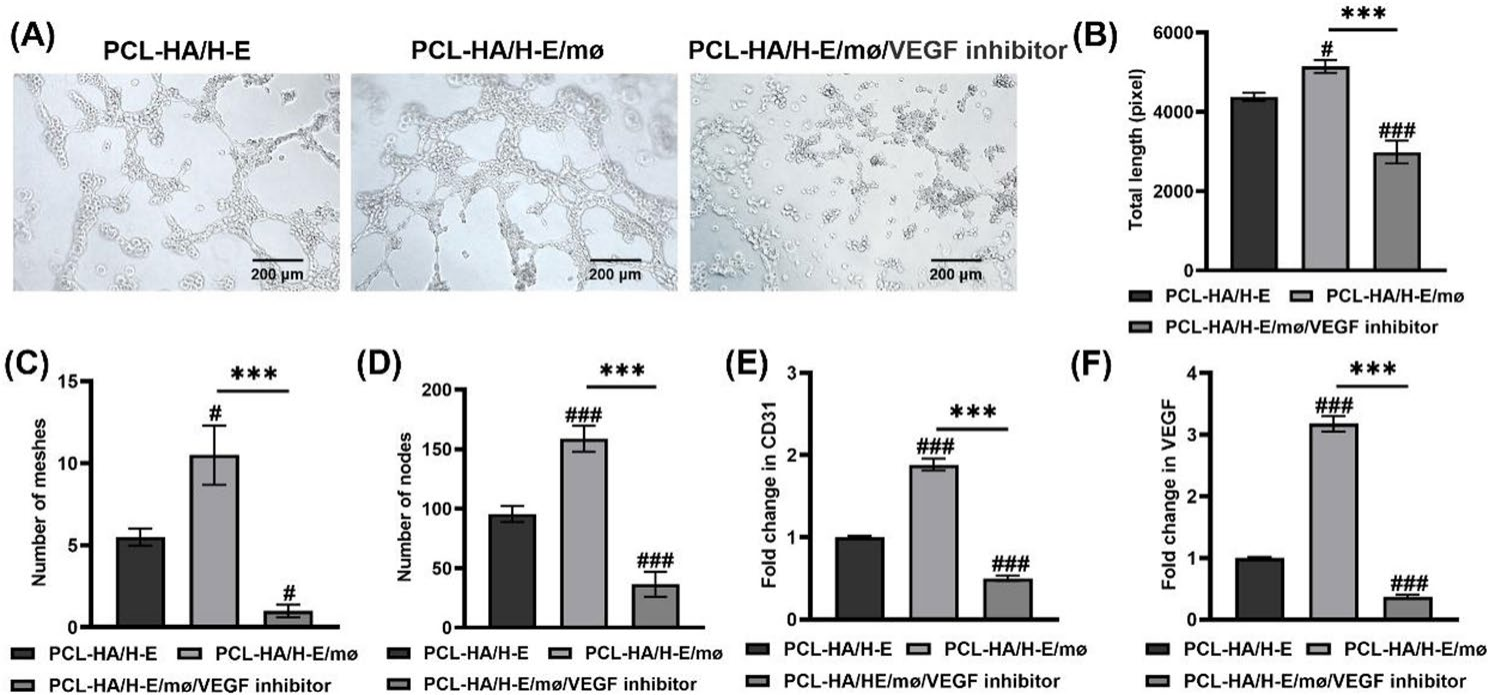
Assessment of the tube formation capability, CD31 and VEGF gene expressions of HUVECs treated with cell culture supernatant collected from H-EMSCs seeded on PCL-HA (PCL-HA/H-E) or H-EMSCs and mø co-seeded on PCL-HA (PCL-HA/H-E/mø) or H-EMSCs and mø co-seeded on PCL-HA and VEGF inhibitor (PCL-HA/H-E/mø/VEGF inhibitor). **A** Tube formation assay of HUVECs in the PCL-HA/H-E, PCL-HA/H-E/mø and PCL-HA/H-E/mø/VEGF inhibitor groups. Scale bar = 200 μm. **B** Quantification of the total length of the tubes formed. **C** Quantification of the total number of meshes formed. **D** Quantification of the number of nodes formed. **E** CD31 gene expression of the HUVECs. **F** VEGF gene expression of the HUVECs. ^***^p < 0.001. ^#^*p* < 0.05, ^###^*p* < 0.001 vs. PCL-HA/H-E group

To further confirm VEGF’s role in promoting greater angiogenesis in the PCL-HA/H-E/mø condition vs. PCL-HA/H-E condition, VEGF inhibition experiments were designed. It was found that the tube formation capability of the HUVECs (total tube length, number of meshes and nodes) was significantly decreased when the VEGF inhibitor was added to the PCL-HA/H-E/mø condition vs. the no inhibitor or the PCL-HA/H-E condition (Fig. 6A and D). It was also observed that the VEGF inhibitor group had lower CD31 and VEGF gene expression vs. the no inhibitor and PCL-HA/H-E groups (Fig. 6E and F).

**Fig. 7:**
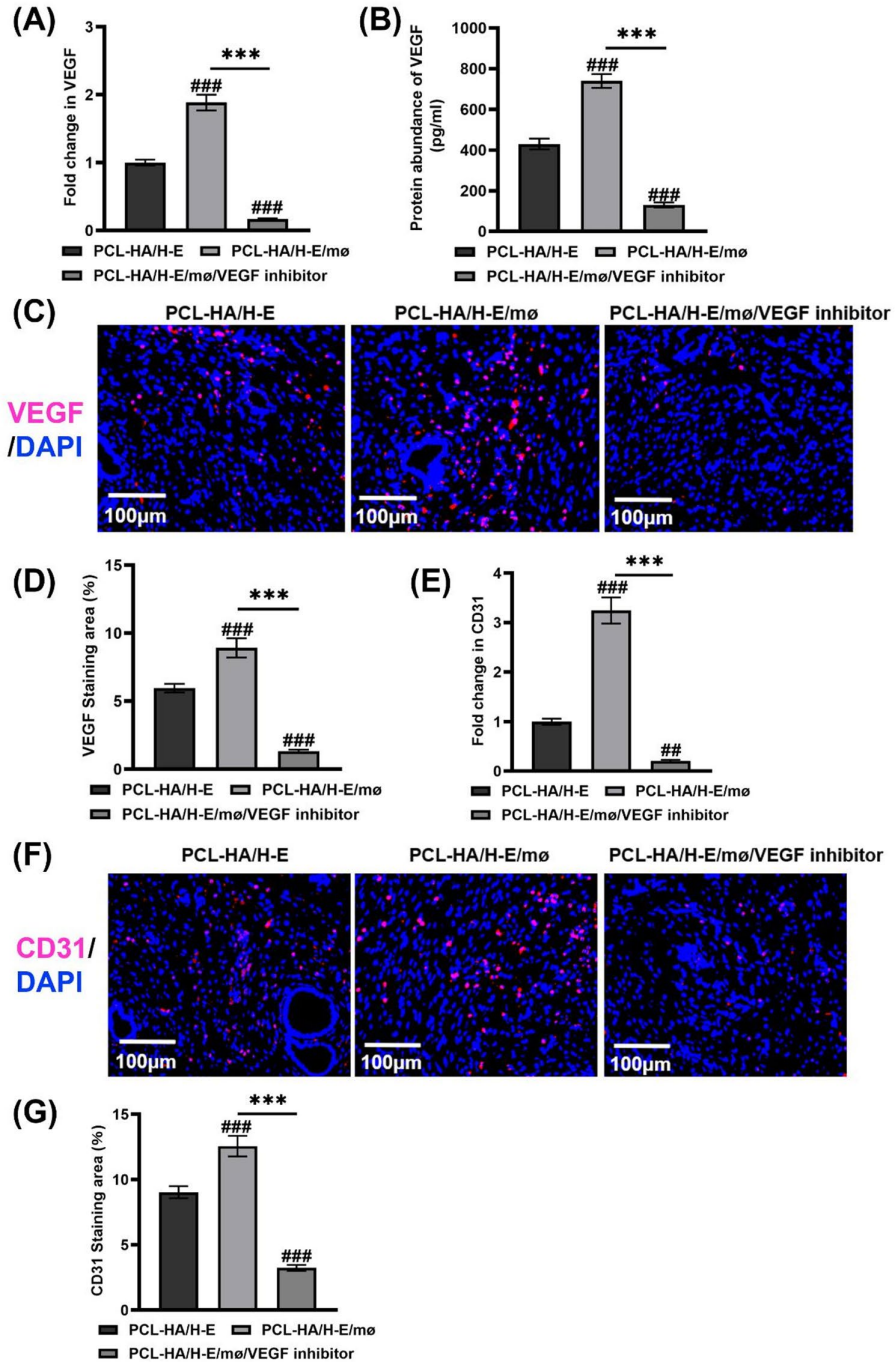
VEGF, CD31 gene and protein expression of rat endometrial tissues of the PCL-HA/H-E, PCL-HA/H-E/mø and PCL-HA/H-E/mø/VEGF inhibitor groups at day 7. **A** VEGF gene expression. **B** Quantification of VEGF protein concentration (pg/ml). **C** Representative images of VEGF immunostaining. Cell nuclei stained in blue while VEGF stained in red. Scale bar = 100 μm. **D** Quantification of VEGF staining area (%). **E** CD31 gene expression. **F** Representative images of CD31 immunostaining. Cell nuclei stained in blue while CD31 stained in red. Scale bar = 100 μm. **G** Quantification of CD31 staining area (%). ^***^p < 0.001. ^##^*p* < 0.01, ^###^*p* < 0.001 vs. PCL-HA/H-E group

**Fig. 8:**
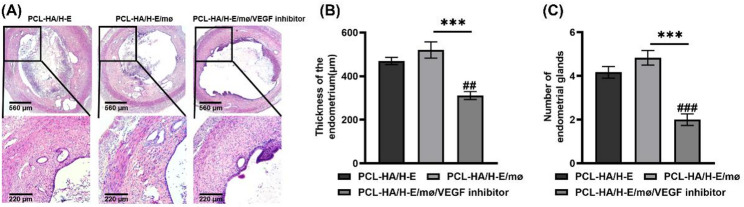
H&E staining of rat endometrial tissues of the following groups: PCL-HA carrier with H-EMSCs mono-delivery (PCL-HA/H-E), PCL-HA carrier with H-EMSCs and mø co-delivery (PCL-HA/H-E/mø) and PCL-HA/H-E/mø with VEGF inhibitor (PCL-HA/H-E/mø/VEGF inhibitor), at day 7. **A** Representative H&E images of the rat endometrial tissues of the different treatment groups at day 7. Scale bar = 560 μm. Enlarged images are shown at the bottom and scale bar = 220 μm. **B** Quantification of the endometrium thickness. **C** Quantification of the number of endometrial glands. ^***^p < 0.001, ^##^*p* < 0.01, ^###^*p* < 0.001 vs. PCL-HA/H-E group

In addition, in the in vivo VEGF inhibition experiments, the endometrial tissues of the PCL-HA/H-E/mø/VEGF inhibitor group showed a significant decrease in VEGF gene and protein expression (Fig. 7A and D), suggesting successful VEGF inhibition in the endometrial tissues. The inhibition of VEGF in the PCL-HA/H-E/mø condition yielded a corresponding significant decrease in CD31 gene and protein expression, which further confirmed that the higher pro-angiogenic effect of PCL-HA/H-E/mø was associated with its higher VEGF production vs. PCL-HA/H-E (Fig. 7E and G). It was also found that with VEGF inhibition, PCL-HA/H-E/mø showed decreased endometrial damage repair outcomes, as evidenced by decreased endometrial thickness and reduced number of endometrial glands (Fig. 8).

## Discussion

Endometrial tissue injury and abnormal endometrial tissue repair (e.g., IUA) are amongst the most important causes of female infertility [35]. The current treatments for IUAs mainly include hysteroscopic lysis of adhesions, hormonal therapy and the use of a physical barrier (for example, balloons) to prevent adhesion [1, 3]. However, those strategies demonstrated low effectiveness especially in patients with moderate or severe IUAs [4], because these approaches only rely on the endometrial tissue’s self-repair capacity rather that promoting innate repair processes during the pathological changes of the injured endometrial tissue. Recent studies investigating the pathogenesis of IUAs have reported that by inhibiting chronic inflammation within the endometrial micro-environment and promoting angiogenesis of the damaged endometrial tissue, it would be possible to enable effective treatment for IUAs [5, 6].

Biocompatible biomaterial-based electrospun membranes, which can serve as an effective cell delivery vehicle that retain cells locally for longer periods, and MSC therapies have gained popularity in recent years towards enhancing the IUA treatment outcomes [36–38]. Amongst the different types of adult MSCs, H-EMSCs attracted great attention for endometrial tissue repair and regeneration as they have been shown to participate in innate endometrial tissue repair during the menstrual cycle, have self-renewal, differentiation, low immunogenicity, low tumorigenicity characteristics, and can be easily-accessed from the adult endometrial tissues [11, 27]. Moreover, mø play important roles in modulating endometrial tissue homeostasis and enhancing endometrial tissue growth [14, 15]. Although both H-EMSCs and mø have great potential in regulating endometrial tissue repair and regeneration, the question of whether the co-delivery of the two cell types on a biomaterial-based electrospun carrier can effectively promote endometrial tissue angiogenesis and repair remains to be answered.

Angiogenesis, which refers to new blood vessel sprouting from pre-existing ones, can be induced by inflammation, wound healing and neoplasia in various tissue types [39–41]. However, it is worth noting that the endometrium is one of the few tissues in human adults where periodic physiological angiogenesis occurs [42]. Insufficient endometrial angiogenesis could increase the resistance to endometrial blood flow from uterine arteries, impair endometrial receptivity during peri-implantation [43], which consequently leads to recurrent implantation failure during in vitro fertilization and embryo transfer [44]. Angiogenesis of intrauterine tissue is of great importance to support the endometrial reconstruction after the menstrual period and prepare a well-vascularized and receptive endometrium for embryo implantation and placentation [5]. In addition, it was found that IUA patients typically have vascular closure and lower microvascular density in their endometrial tissues [8, 45], emphasizing that angiogenesis in the endometrial scar regions is important for effective endometrial repair. However, there are very few studies in the IUA field that have investigated strategies to promote endometrium angiogenesis and therefore fundamentally enhance endometrial repair and regeneration.

In this study, it was observed that the unique combination of PCL-HA electrospun system with H-EMSCs and mø co-delivery (PCL-HA/H-E/mø) significantly increased the endometrium thickness at days 7 and 14 in the endometrial damage model vs. the NR (normal repair) and PCL-HA membrane alone groups. The implantation of PCL-HA/H-E/mø also restored the number of endometrial glands vs. NR or PCL-HA alone. In investigating the effects of the H-EMSCs and mø co-delivery system on promoting angiogenesis in the damaged endometrial tissue, it was significant to observe that PCL-HA/H-E/mø enhanced more CD31 gene and protein expression indicating more angiogenesis occurring at days 7 and 14 post-implantation, when compared with PCL-HA/H-E, NR or PCL-HA alone. Similarly, the angiogenic marker VEGF gene and protein expression was also higher in the PCL-HA/H-E/mø vs. the other conditions. The findings in this current study suggested that biocompatible PCL-HA electrospun membrane for co-delivery of multiple cell types associated with endometrium repair, specifically H-EMSCs and mø, could have great potential in boosting endometrial angiogenesis and promoting endometrial tissue repairing outcomes (increased endometrium thickness and number of endometrial glands).

The potential use of MSCs for enhancing angiogenesis and treating IUAs has been reported previously. For example, H-EMSCs that were induced to have elevated expression of CYR61 were transplanted into a rat uterine injury model, and it was found that those cells induced angiogenesis that further supported the restoration of a full-thickness endometrium [46]. In addition, the exosomes derived from the bone marrow MSCs were found to be able to promote endometrial angiogenesis that further reversed endometrial fibrosis [3]. Previous studies have also tried to use biomaterials as carriers for MSCs or their exosomes to enhance tissue angiogenesis. For example, in a rat IUA model, it was found that human adipose MSCs carried on an acellular human amniotic membrane can effectively improve endometrial angiogenesis [47]. Moreover, adipose MSCs’ exosomes embedded in a Pluronic F-127 hydrogel were demonstrated to significantly accelerate wound healing by boosting angiogenesis and the ECM remodeling [48]. The literature also supported that, mø, in particular those that express VEGF receptor 1, can promote endometrial angiogenesis [14]. All these previous results aligned well with the findings of the current study that the co-delivery of mø and H-EMSCs supported the endometrial angiogenesis and tissue repair to a greater extent compared to mono-delivery, biomaterial alone and NR groups.

In exploring the mechanisms underlying the enhanced CD31 gene and protein expression (which indicated enhanced angiogenesis), supported by the co-cultured patch (PCL-HA/H-E/mø), it was found that the elevated VEGF production at the gene and protein levels was one of the potential factors that contributed to the enhanced angiogenesis in the co-culture condition. In addition to the VEGF signaling needed to promote angiogenesis, as evidenced in this study for endometrial tissue repair, studies have also reported that H-EMSCs and perivascular MSCs can enhance angiogenesis via AKT/ERK and HIF1α-dependent pathways, respectively [11, 49]. However, whether those signaling pathways are involved and operated effectively when the cells are co-delivered on a synthetic/HA composite, i.e. the PCL-HA/H-E/mø co-culture system used here, to promote endometrial angiogenesis remain to be explored in further studies.

## Conclusions

To conclude, this study successfully developed a rat endometrial injury model and a PCL-HA based H-EMSCs and mø co-delivery system. The co-delivery system significantly enhanced endometrial angiogenesis and enhanced endometrial damage repair (evidenced by increased endometrial thickness and the number of endometrial glands) vs. the H-EMSCs mono-delivery system. VEGF was one of the factors that was identified to play a significant role in inducing endometrial angiogenesis in the co-delivery condition. This study provided significant insights into using the co-delivery of H-EMSCs and mø on a composite biomaterial platform for effectively inducing endometrial angiogenesis and potentially enhancing IUA treatment outcomes.

## Electronic Supplementary Material

Below is the link to the electronic supplementary material.

Supplementary Material 1: Table. 1 Clinical information of patients who donated endometrial tissue samples of the study.

## Data Availability

All data generated or analyzed during this study are included in this article and its supplementary information files.

## Abbreviations

IUA: Intrauterine adhesion
H-EMSCs: Human endometrial mesenchymal stem cells
Mø: Macrophages
PCL: Polycaprolactone
HA: Hyaluronic acid
NR: Normal repair group
ERK: Extracellular signal-regulated kinase
MSCs: Mesenchymal stem cells
ECM: Extracellular matrix
FDA: Food and Drug Administration
SD: Sprague-Dawley
HUVECs: Human umbilical vein endothelial cells
ANOVA: Analysis of variance
