# Supplementary material for: Co-delivery of endometrial mesenchymal stem cells and macrophages by an electrospun patch promotes angiogenesis during endometrial injury repair via VEGF related signalling
Source: Stem Cell Res Ther. 2026 Feb 16;17:108. doi: 10.1186/s13287-026-04929-2 (PMC12988638; doi:10.1186/s13287-026-04929-2)
Supplement: Supplementary file 1 — Supplementary Material 1: Table. 1 Clinical information of patients who donated endometrial tissue samples of the study. [file 13287_2026_4929_MOESM1_ESM.docx]

**Supplemental Table 1**. Clinical information of patients who donated endometrial tissue samples of the study.

| **Patient No.** | **Age (years)** | **BMI** | **Health condition** | **Menstrual cycle stage** | **Collected tissue type** |
| --- | --- | --- | --- | --- | --- |
| **1** | 33 | 19.6 | healthy | menstrual cycle day 2-4 | endometrial tissue pieces |
| **2** | 35 | 22.1 | healthy | menstrual cycle day 2-4 | endometrial tissue pieces |
| **3** | 28 | 23.9 | healthy | menstrual cycle day 2-4 | endometrial tissue pieces |
| **4** | 25 | 19.6 | healthy | menstrual cycle day 2-4 | endometrial tissue pieces |
| **5** | 26 | 24.2 | healthy | menstrual cycle day 2-4 | endometrial tissue pieces |
| **6** | 26 | 21.9 | healthy | menstrual cycle day 2-4 | endometrial tissue pieces |
